# Online Interactions: Mobile Text-Chat as an Educational Pedagogic Tool

**DOI:** 10.3390/bs12120487

**Published:** 2022-11-30

**Authors:** Pingping Huang, Qianyun Yu

**Affiliations:** 1 School of English for Specific Purposes, Beijing Foreign Studies University, Beijing 100089, China; 2 Institute of Education, University College London, London WC1E 6BT, UK

**Keywords:** online interaction, pedagogy, mobile learning, distance education, conversation analysis

## Abstract

The reactions of education systems to the global lockdowns implemented during the COVID-19 epidemic highlighted that there remain questions regarding how everyday technologies might be used to support mass education. This paper draws on Conversation Analysis in online textual communication to study key features of mobile text communication by analysing book discussions among adult students of an online reading programme. We captured and analysed three patterns of interaction (i.e., single linear conversations; intertwined conversations; trunk-branch conversations) as to their affordances for educational communication. This study shows that synchronous text has distinctive communicative features, including short text exchanges and various turn-taking patterns, which are different to the elaborated forms of discourse expected in schools. Though “disorder” and “messiness” accompanied the interactions, we take them as opportunities rather than challenges of education and suggest that appropriate pedagogic design may enable teachers to utilise this distinctiveness to develop various learning environments.

## 1. Introduction

The mass closure of educational institutions around the world during the COVID-19 epidemic left educational institutions scrambling to find technologies that could afford distance learning. UNESCO provides a list of possible resources that may help support education, which includes a wide range of technologies including mobile learning tools and resources. Mobile technologies have long been seen as offering important resources for providing “ubiquitous” learning that stretches beyond the classroom or specific physical locations [1,2]. These tools are seen as providing “seamless interactions with people who may be geographically distant, sharing and creating of images and videos of common interest, and engaging in activities of interest to the user.” [3] (p. 82). High levels of ownership and increasingly global internet access mean that mobile phones in particular are potentially important resources for schools [4] and of key relevance in the context of moments of crises such as the COVID-19 epidemic.

Chat-based instant messaging applications that afford interaction between groups of people are of particular relevance for constructivist pedagogies [5,6] and have been shown to have positive effects both in schools [7,8] and in adult learning contexts [9,10,11]. Burden et al. [3] systematic review of mobile pedagogies found evidence of innovations that would be impossible without mobile technologies, highlighting the distinctive contribution that they can make to learning.

However, their levels of uptake both within the classroom and as a resource for work outside of the classroom remain patchy at best [3]. This is not surprising given the substantial issues that such technologies raise from an education perspective. The United Nations noted the risks of relying too heavily on these technologies as a replacement for classroom teaching, emphasising the need for diversity [12]. Further, mobile technologies require additional technical skills on the part of all users and present real challenges in terms of privacy and data security [13]. Particularly relevant to the topic of this paper, such technology can radically alter the nature of social interaction in a distance learning setting [14], changing the ways that students interact with each other, with the teacher, and with learning materials and content.

From the perspective of Conversation Analysis (CA), the analytic framework for this paper, interaction and communication are regarded as the basis of all activity and of people’s understanding, so changes in how communication works fundamentally alter the nature of the activity at hand. Following on from this, to understand how mobile technology impacts learning we need to look closely at the micro-practices of communication of real people in real learning scenarios. Kim [15] has argued that the vast number of studies in this area tend to focus on learners’ cognitive development, neglecting the interactional features of the learning environments themselves. Studies of synchronous mobile learning have explored diverse themes, such as learners’ academic achievement/improvement like vocabulary gain [16], geographical fieldwork [17], language proficiency [18], learners’ satisfaction [19], and levels of contribution to intercultural discussions [20], but in most cases, these explorations occur without a close concern for interaction. Our analysis aims to plug this gap by showing some of the key patterns found in a study group using synchronous mobile conversations.

## 2. Mobile Technology in Education: Features and Uses

Mobile communication technologies constitute new forms of interaction, and substantial interest is developing in the semiotic and interactional practices that comprise them as well as their role as tools for formal education in schools [7,8] and professional training such as nurse education [21]. The move to using technology for collaboration involves changing the resources that people have at their disposal to access information and communicate with one another. A large body of work is developing around how new text and video “chat” technology is influencing people’s communicative practices. The majority of studies of communicative action through technology have occurred in non-educational contexts [22] such as business communication [23]. Starting with asynchronous communication, research has shown that when using tools such as discussion boards, text messages or other non-real-time chat messages, participants often use postings to achieve multiple conversational/communicative actions. So, they may greet fellow members, introduce themselves, and make requests all within the same conversational “turn” [24]. This is very different to spoken or synchronous written interaction where such actions tend to occur at sequentially distinct moments. Relatedly, the topics of conversation are often initiated in very distinctive ways, and can essentially begin at any point. This is also remarkably different in co-presence or mediated real-time communication [25]. Because of the above features, asynchronous communication has famously been characterised as “disorderly” [26,27] and highly complex [28] and users make use of things such as “addressivity” and distinctive lexical/grammatical structures to combat this. 

The communicative possibilities offered by mobile technologies are relative to their specific properties. The term “affordances” refers to the ways that technologies give rise to certain possibilities of action as a result of the conventions of practice that people employ when using them [29]. Shirley and Shafirova [30] looked at how students in an online postgraduate course using WhatsApp constructed the roles of “expert” and “novice”, and the affordances of the technology in allowing users to re-read messages and to have different sequential conversational threads. In terms of communicative practice, the particular interface being used, the ways that a keyboard is laid out, the time lag between posting messages and other users receiving them, the ways the interface allows users to use communicative tools such as voice, emoticons, GIFs, weblinks and so on: all of these make possible particular kinds of interaction, while restricting others. The different communicative modes within a technology may offer distinctive interactional affordances.

Mobile technology has been shown to have a positive impact on second/foreign language reading performance such as EFL reading from students’ learning outcomes [31] and teachers’ perspectives [32]. However, very little attention has been directed toward mobile-assisted first language reading practices in Chinese language contexts [33]. Chang et al. [33] research is one of the few exceptions here, the authors found that students of lower ability in Chinese language comprehension showed greater improvement when they used the online system than students who did not. While the findings of the study are interesting, there are significant methodological limitations of this study. The study was based on self-report questionnaires which do not enable us to understand people’s practices of learning as social actions, and have limited value in reflecting on the praxis of learning. Technically, Chang et al. [33] research is also limited as the technology did not contain chat functionality, which restricted its use as a genuinely collaborative tool. Our study aims to address both of these methodological limitations by looking at how real-time chat technology is used by participants as a discursive tool in particular.

It is noted earlier that CA is an established approach to the study of the social accomplishment of learning as a communicative practice [34]. This research has looked at diverse topics including issues such as the development of the topic organisation [25,35], the integration of new speakers into a conversation [36,37], the repair of mistakes [38], and other interactional problems [39]. Real-time synchronous and “quasi-synchronous” chat is a substantial area of new interest here [40,41], and we aim to use, apply and develop the conceptual tools that have already been used to analyse communicative actions in educational environments. In this article, our interest is in looking at the conversational features of communication that are used to achieve the social actions constitutive of discussing reading in WeChat, an instant messaging and social media app. We draw on the analysis to describe the general features of interaction focusing particularly on the turn-taking sequences that were present in the chats and the pedagogic implications arising from them.

## 3. Research Methods

The study explores a reading programme delivered by an educational technology company based in China. The programme aims to encourage people to form a habit of reading and help them to overcome problems such as a lack of desire to read or low reading proficiency. The main target group are adults aged from twenty to forty, who are able to make use of the fragmented off-work time to participate through mobile phones. The participants joined the programme voluntarily and described various motives for participation: some of the participants referred to an absent-mindedness in reading, some found difficulty in choosing books, while others felt that they needed the opportunity for discussion with others and a community to motivate them to keep reading every day.

Over ten months, learners read selected sections of 40 books covering nine reading themes and are encouraged to spend 15 min reading every day. According to the syllabus, each month users are offered sections of four different books to read under the themes including self-management, communication and cooperation, psychology, logical thinking, science, philosophy, society, art and business attainments. New reading content is unlocked daily, and participants are placed into groups based on location and are encouraged to discuss or share questions and reading experiences with other group members through WeChat groups. Discussion in the WeChat group is voluntary rather than compulsory. Self-study readers may choose not to join a WeChat group or keep silent from the beginning to the end. They all got e-certificates to prove their participation once the programme was finished. WeChat is the most popular chat application used in China. It is a powerful tool that allows sending texts, images, emojis, videos, document files, web links, location maps, making voice and video calls and even transferring money. 

We recruited 55 participants from the programme who were organised into two separate groups. Table 1 demonstrates their general background information in terms of age, education level and status of employment. Participation was entirely voluntary and users were given a project information sheet explaining the methods and aims of the study: those who agreed to participate gave written agreement before being assigned to a group. Two of the researchers acted as moderators for the groups in the first instance, after which the members chose weekly moderators from the groups. The main role of the two researchers was to encourage group discussions by initiating questions and participating in discussions. Once participants started to discuss, researchers usually tended not to play the dominant role but noted down how the discussion went. Over eight months, the researchers took screenshots of their mobile devices in WeChat to capture the conversations. 

The researchers transcribed and translated Chinese conversations into English and analysed them through conversation analysis. A total of 3178 turns from the two groups were recorded (see Table 2). A large percentage of the turns that included text (i.e., not just emoji or images) are short turns (62.47% of all the posts). Here, we define “short turns” as posts with text between 1 to 21 Chinese characters (about one line on tablets and 1.5 lines on smartphones).

Drawing on the literature in conversation analytic studies of pedagogy, the analysis involved providing a detailed account of the types of action undertaken in the chat (see Table 3). CA has shown that a common feature of educational interaction is “triadic dialogue”, involving a three-part Initiation-Response-Feedback/Evaluation (IRF/E) sequence [42,43,44]. In classrooms, teachers often initiate a question-answer sequence (initiation), which students then answer (response), with a follow-up third turn by teachers that evaluates/gives feedback on the answer (Feedback/Evaluation). We looked for sequences that had some resemblance to these types of actions, as well as to other common sequences such as “questions with known answers”, where participants ask a question not to find out the answer (which they already know) but to encourage some other kind of action, such as testing the other’s knowledge [34].

**Table 1:** General information of research participants.

Age			
18–29	30–39	40–49	Others
58%	34%	7%	1%
**Education**			
Undergraduate	Graduate	Others (secondary education, etc.)	
74%	11%	15%	
**Employment**			
Full-time students	Employed	Unemployed/self-employed	
9%	85%	6%	

**Table 2:** Size of data and features of turns.

	Number of Participants (Exclude Researchers)	Number of Turns in Total	Turns that Contain Text	Short Text Turns
Group 1	27	1676	1450	852 (58.76%)
Group 2	28	1502	1316	876 (66.57%)
Total	55	3178	2766	1728(62.47%)

Based on this analysis (see Table 3), we tracked the initiation and response, then teased out the connections among turns with the help of drawing figures showing interactional relations between turns. We, therefore, identified four main patterns that happened in the online conversation, which are “single linear interaction”, “intertwined interaction”, “trunk-branch interaction”, and “no response”. The category of “no response” refers to the initiator giving a comment or sending an emoji/image/link without receiving any feedback or response from group members. 

**Table 3:** Coding framework for educational actions.

	Moderators	Participants/Users
Asking a question to the group		
Asking a question to an individual participant		
Asking a question to a moderator		
Giving feedback to individuals		
Giving feedback to the group		
Answering a question directed to an individual		
Answering a question directed to the group		
Asking ‘question with a known answer’		
Initiation-Response-Feedback (IRF) sequence		

## 4. Analysis

As shown in Table 4, “intertwined interaction” presents the highest frequency of occurrence among the four identified patterns, which is followed by “trunk-branch interaction” and “single linear interaction”. The following sections concentrate on each of the three distinctive patterns of interaction, showing the interactional characteristics that each possessed, and reflecting on the pedagogical implications of these differences for education. 

**Table 4:** Occurrence of patterns.

	Single Linear Interaction	Intertwined Interaction	Trunk-Branch Interaction	No Response
Group 1	31	47	36	18
Group 2	21	51	48	15
Total	52 (19.5%)	98 (36.7%)	84 (31.5%)	33 (12.3%)

### 4.1. Single Linear Interaction

In Extract 1 (see Table 5), two participants were discussing a recent viral video shared by Jane showing an experiment to test whether children would accompany an adult that they did not know. It was initiated based on a participant’s comment on a reading chapter concerning the differentiation of correlational relationship and causal relationship and the reliability of conclusions. After this, participants discussed the validity, reliability and ethics of the experiment in the video in Extract 1 (Table 5). The conversation comprises five consecutive turns, starting with an utterance by Jane.

In Turn 657 and 658, Jane shared a popular video that showed a social experiment on whether children would go off with a stranger. Jane asked an open question about the group members’ opinions about showing the video to children. Qing joined the discussion in Turn 668 producing a turn that suggested that the video would frighten children, and then asked the group how children could trust other people in the future. They then included a turn expansion “just my personal opinion”, which is the kind of utterance that has been shown to follow turns that implicate an epistemic disalignment between participants [45]. After a two-minute pause, Jane took up this question replying that many people did show it to their children and that they would like to replicate the test with their children. Qing replied immediately afterwards with three turns that each expanding on her original argument in Turn 668, elaborating reasons why the video would not be healthy, concerning learning these ideas from a psychologist. Following these turns and a short three-minute pause, Jane replied (in Turn 674) saying that she agreed that feelings of danger are negative.

**Table 5:** **Conversation Extract 1** (Information in the brackets is added by researchers to aid understanding of the translation).

Turns	Name	Content	Length in Chinese (Characters)
1:27 p.m. (China time)			
667	Jane	Will you show the video to the children	14
668	Qing	It’ll frighten them too much, how can they face others and trust other people in the future?	28
669		I wouldn’t, just my personal opinion.	9
1:39 p.m.			
670	Jane	But the reality is that many parents show this video to their children straight after seeing it, or plan to do the same test to see if their own children go off with a stranger.	51
671	Qing	Infinitely over-emphasising a certain danger awareness is also a disastrous thinking pattern, I think the mental status (of children) won’t be healthy (if you do that). That is what I learned from a psychologist. Even if the social environment is not safe, it is not necessary to over-emphasise danger like this.	71
1:44 p.m.			
672		The problem is, if children know it’s just a test, it won’t have real effects. If children are tested without knowing it, how to remove the psychological shadow this test brings to them?	63
673		We’re not psychologists; we’re really not able to know how much influence there is in it.	26
1:47 p.m.			
674	Jane	I agree, it’s terrible if everyone feels that they are in danger.	14

The turns have a clear interactional relationship to each other and each deals with the implicatures of the previous action; in this way, these turns reflect similar sequential orders to those found in dyadic face-to-face or mediated synchronous classroom interaction: each turn shows its interactional relevant to the preceding one, and are produced in many cases with a minimal time gap between each post [46]. However, we also see gaps in messages of a minute or more that are longer than one would typically find in face-to-face classrooms. Despite these gaps, the interaction proceeds as if there had been no gap, with none of the mediating actions that people normally produce following “extended” pauses.

### 4.2. Intertwined Interaction

In the context of online discussion with more than two participants in a group, turn-taking is frequently very different to the pattern outlined in the previous section. Extract 2 (see Table 6) shows a discussion between five participants (including one of the researchers—R1). This extract includes two embedded conversational sequences. Turns 151 to 155 can be read as a sequential exchange relating to the notion of inherited schemes of understanding. At Turn 156, Congcong posted a picture taken from his phone, but at Turn 157 Wen continued the initial discussion. At Turn 158, Congcong provided a textual expansion of his picture by explaining that it showed the rain he was facing on his way to work. This results in a series of responses from the other participants, including R1, Dong and Wen. Therefore, a single linear conversation pattern is no longer workable. Instead, there are two streams of conversations intertwined in this extract.

One point to note is the way that Congcong’s post of a picture at Turn 156 and his subsequent written expansion of it (Turn 158) results in a type of topic change that is unusual in spoken conversation. Button and Casey [47] have shown that where it is self-initiated (as opposed to being initiated by someone else), the topic shift is generally either thematically connected to preceding turns (i.e., by showing how a new topic links to the old one), or it involves interactional work such as leaving pauses to create topical “breaks”. Here, however, Turns 156 and 158 create a topic shift without an orientation to the existing chat.

**Table 6:** **Conversation Extract 2**.

Turns	Name	Content	Length
		10:05 p.m.	no. of characters
151	Wen	(I) read your own extension to the readings in this group, especially the diffusion of responsibility mentioned by Dong, I find it interesting. As for the issue of conformity mentioned in today’s reading, I think, there’re deep-rooted historical cultural reasons for those conformity behaviours known to all in China, i.e., as the texts say: identification and internalisation.	96
152		Like “everyone sweeps the snow in front of their own doors”, one point is that the behaviours of ancestors inform the identity of future generations; another point is that the long-lasting cultural background makes people have internalized mind-set (become inured to the unusual, and so behave the same way as their ancestors and most other people)	80
153	R1	One’s children should do what other children do, they must not become weaker by comparison. One should own what other people own. Conformity, haha, actually also comes from comparing oneself with others	45
10:14 p.m.			
154	Wen	Yes, haha, (it relates to) the influence of family. Actually, when children are shaping their worldviews, every behaviour that the family brings to them will be internalised unconsciously and integrated into their sub-consciousness, handed down from generation to generation	70
155	Dong	Ultimately, we are social animals and we conform in order not to be isolated	21
156	Cong-cong	[picture]	1
157	Wen	Chinese thinking patterns are like a mosquito coil and Americans have a linear thinking pattern, which is probably an inheritance and just involves following social ideology.	35
158	Cong-cong	Heavy rainstorm, (I have to) go to work…tears 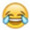	13
159	R1	Typhoon weather 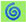 , be careful	8
160	Wen	Nigh shift? Or do you work overtime? 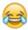	9
161	Dong	Come on, (let me) give you a hug 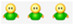 @Congcong	14
162	Cong-cong	Overtime working…my boss got me into a fix, call male staff urgently to work overtime…in order to guarantee the use of electricity for you, life is not easy for us. 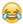	38
163	Wen	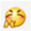 Dong, you summarise so well~ in the ancient time, isolation means decreasing the possibility to survive, while living in groups is beneficial for survival, so (people) follow the crowd, which is based on the irreplaceability of survival.	57
10:20 p.m.			
164		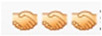 salute! Be careful! @Congcong	18
165	Cong-cong	(I) go to work first and discuss with you guys tomorrow 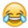	13

Continuing with Extract 2 (Table 6), at Turn 163, Wen returned to the previous conversation, “interrupting” the newly initiated conversation. This kind of shifting back and forth between different topics is referred to in online textual conversations as “disrupted adjacency” [27]. Researchers have shown that one of the common strategies used to deal with this difficulty is to use “addressivity” [36,37], i.e., to refer to participants directly in their contributions to make clear who their comments are directed towards (i.e., the “recipient design” of the turn). In Turn 163, Wen referred to Dong directly at the start of the turn but in the subsequent Turn (184) they addressed Congcong at the end of the turn using an “@” symbol before their name. This practice of naming is distinctive to written interaction, and is a key resource to being able to maintain the “reading path” of the text [27,40].

**Figure 1:**
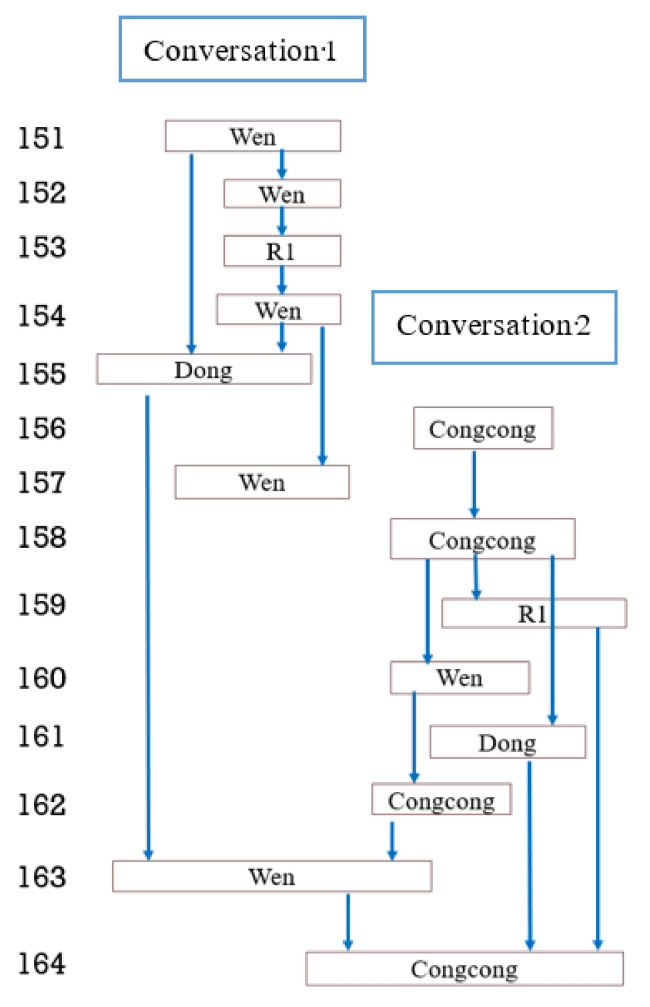
Intertwined conversation pattern as represented in Table 6.

Figure 1 depicts the intertwined relationship between the two conversations initiated by Wen and Congcong, respectively, and the turns shown in Extract 2 (Table 6). It helps to visualise the ways that the exchanges are conducted back and forth in an online group discussion.

It can be seen from the above that where conversations involve more than two participants, the conversational structure becomes more “disorderly” [27,48]. Even groups as small as four, as depicted in Extract 2 (Table 6), can show substantial “messiness” in the organisation of turns. To be clear, to refer to “disorder” means that there is likely to be an increase in interactional phenomena such as questions not being answered; contributions with an unclear topical referent; frequent change in the topic; or the production of multiple topics of conversation at the same time. The more participants there are in a group, the more likelihood of conversational disorder there may be. However, it is not the intention of this research to make any prior judgements about what is appropriate or otherwise to any given context—rather the point is simply that in designing educational tasks with mobile technology, instructors and participants should be mindful of the kinds of phenomena that are likely when certain group sizes are used. 

### 4.3. Trunk-Branch Interaction

The third type of turn-taking found involves the production of multiple responses to a single post. Extract 3 (see Table 7) provides a typical example. Daojian shared a mind map to summarise reading content which led to a set of consecutive responses between Daojian and Dong (Turns 47–51). If it is read sequentially Daojian’s smiley emoji at Turn 51 could be treated as a response to Dong’s emojis in the previous turn. However, there is ambiguity here as it could also be a continuation of Daojian’s contribution from line 49. This type of ambiguity is not insignificant in understanding textual interaction. There are serious questions to ask about the impact of such ambiguity on participants’ experience of learning [13]. We will return to this point at the conclusion of the paper.

**Table 7:** **Conversation Extract 3**.

Turn	Name	Content	Length
9:24 p.m.			
44	Daojian	[picture: a mind map]	1
45		You guys can try Xmind to summarise	12
9:30 p.m.			
46		Summarising every day, improving every day	13
47	Dong	Wow, you know how to make mind-map, awesome	15
48	Daojian	It’s easy	4
49		It’s necessary for people who do operations	9
50	Dong	(I need to) lull my children into sleep first, (will) consult you later 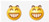	16
51	Daojian	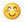	1
52	Nichang	@Daojian Brilliant 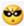	9
53	Jane	@Daojian 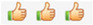	8
54		(I) gain (new) knowledge	3
55	Daojian	You guys can download an Xmind on computer, it’s easy	17
9:33 p.m.			
56		We need to summarise and share our ideas	10
57		[Picture: screenshot of Xmind app on the app store]	1
58		(You) can download this App on your phone	10
59		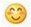	1
60		It’s easy (to download)	4
61	Nichang	An idol has been born 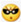	7
62		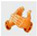	1
63	Jane	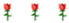	3
64	R1	This will be useful for weekend’s quiz! And (we) can use it in Saturdays for reviewing~	27
9:45 p.m.			
65	R2	@Daojian Wow, do you make mind-map every time you read	18
66	Daojian	No	3
67		But in my work, I use mind maps a lot	12
68	R2	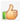 You’re experienced	5
69	Dong	Let’s go and download one	6
9:55 p.m.			
70	Yixiao	The branch (in the mind map) can’t be deleted, speechless	10
71	Daojian	It can, read the help file	10

Continuing with the extract, Nichang and Jane both provided responses that were readable as commentaries on the topic initiated by Daojian. These two turns use “@” to address Daojian directly, with both Nichang’s “brilliant” (Turn 52) and Daojian’s thumbs-up emoji functioning as affiliative relations to these turns Affiliative ‘assessments’ such as these in spoken conversation have been shown to have distinctive “sequential orders”, with recurrent patterns of use in terms of when and how they are produced [49]. The use of the addressee marker “@” shows that the participants treat addressee as a matter requiring interactional work—i.e., that they need to include these markers to make obvious who their evaluations are addressed to. 

Continuing with the interaction, after a brief pause Daojian followed up his comment that the app can be downloaded on a computer (Turn 55) to say it can be downloaded on a phone (Turn 58), with Turn 55 implying that they could use the app as a team for their collaborative work. Following this, participants produce a series of further evaluations (Nichang at 61/62, Jane at 63, R1 at 54, R2 at 65. R2 followed the evaluative “wow”, R2 asked if Daojian made mind-maps every time they read, which Daojian replied to in the next turn and R2 replied with further evaluation in 68). Again, the consecutive sequentiality of these three actions makes them readable as a sequential question-answer action. Following this, Dong proposed to go and download the software (Turn 69) and Yixiao (Turn 70) made a comment about the functionality of the app, which is potentially readable as a complaint that Daojian replied to with an affirmation that the app can do what Yixiao would like.

**Figure 2:**
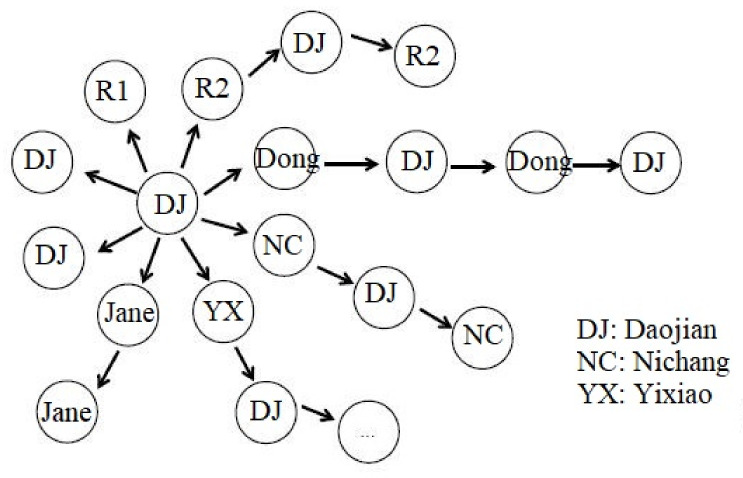
Sample of web-like patterns: multiple responses to a single post.

**Figure 3:**
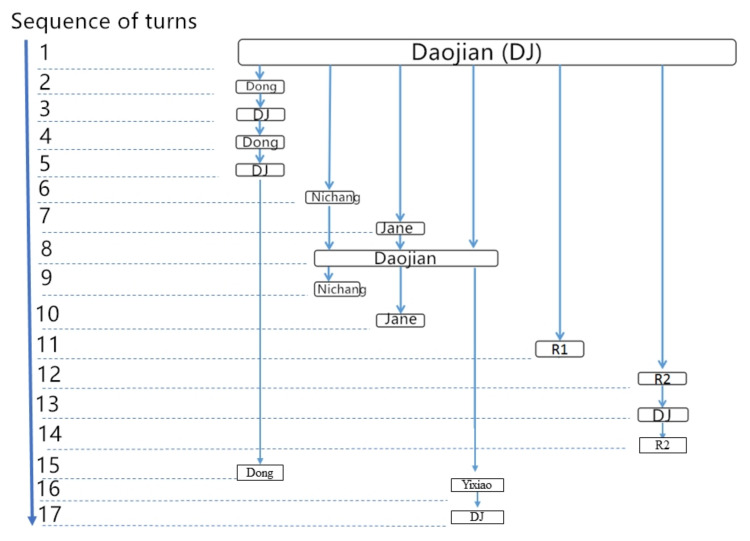
Trunk-branch pattern.

One way to make sense of this sequence is to read it as a set of separate turn responses, which address the initial post (web-like pattern, See Figure 2 for a visual depiction of this). Previous research has applied a similar web-like pattern to visually present features of interaction in non-linear turn-taking [46]. However, this arguably over-simplifies a complex structure that involves greater ambiguity between posts than the model in Figure 2 implies, that it only shows streams of interaction without indications of turn-taking sequence. It tells the interactional relationship between interlocutors without showing conversations in order. Therefore, to improve this visualisation model, we propose a new pattern, the trunk-branch pattern (see Figure 3), meaning that the branching conversations derive from the same trunk, in which the relationship between the sequence of turns, streams and posts is much clearer. 

## 5. Discussion

The features of the interaction that mobile chat technologies (WeChat in this research) offer can be harnessed for distance learning tools. This study investigates three patterns of conversation that happened in the online learning platform, which are single linear patterns, intertwined interactions and trunk-branch patterns. It is concluded that linear sequences of action are more commonly seen in pair discussions with only two participants in the chat group, which is similar to those found in spoken interaction. When more than two participants joined, the “disorder” or “messiness” occurs due to multiply layers of conversation that can happen at the same time. To reiterate, we do not use these terms evaluatively. We saw that participants could use the functionality such as “@”, but also simply naming participants to establish addressivity and to maintain the “reading path” of the interaction. Where turns did have an interactional relevance in consecutive turns, then addressivity was not used, showing that participants only used addressivity to solve particular interactional problems. 

The ubiquity of mobile tools and chat applications makes them a potentially valuable resource for distributed/distance learning. We argue however that it is important to understand in detail how such tools impact the interactional phenomena of “doing learning”. Our analysis aims to concentrate on the relationship between their conversational/communicative features and the implications of these for distance learning pedagogies. While the ordered single linear pair discussion is needed in educational instructions, it does not mean that “disordered” interactions are unwanted. Instead, different interaction patterns serve different pedagogic activities and finally contribute to supporting a diverse educational environment. Text chat is a genre-specific form of writing, and it is not always organised in the same way as speech or as more formal academic prose. It could be problematic to treat these genre-specific features as an unwanted departure from more conventional forms of spoken or formal written communication. Instead, it may be more beneficial to make use of these aspects and to treat them, as users do, as useful devices for achieving coherent text talk.

The pedagogic implications are intended to take account of the core findings that this study has produced related to the organisational features of interaction via text-chat. In particular, they aim to manage the online educational interactive environments that can arise from multi-party discussions in relation to overlapping and “messiness” in the dialogue. The intertwined pattern as shown in Extract 2 (Table 6), for example, could be categorised as “unexpected” contributions and “off-task” conversation in group discussions since the topic has been shifted to the heavy rain which is not directly related to reading content [50]. Some teachers may signal “unexpected” contributions as problematic [51] and do not appreciate “off-task” conversations [52]. However, such talk can still yield necessary learning opportunities [51] rather than being “unwanted”. Conversations can have a range of goals such as knowledge/opinion/information sharing and rapport building [53], which applies to educational contexts as well. The former fulfils the transactional or cognitive learning purposes, while the latter meets social purposes. Vygotsky [54] acknowledges learning as an essential social activity rather than a purely cognitive process. Bakhtin [55] notes the importance of pluralistic utterances in peer interactions. Conversations for social and affective purposes will contribute to the accomplishment communication such as building rapport and releasing emotion [56]. As argued by Markee [57], “off-task” conversation does not equate with not “on-task” and it is naturalistic and closer to interactional needs of learners in real life. Allowing “off-task” conversation, to some extent, encourages learners’ creativity in the learning process. Therefore, teachers are expected to view unpredictability of students’ interactions as valuable contributions and enable the fulfilment of interaction for social purposes to benefit academic and cognitive learning. 

Teachers may modify online pedagogic designs to confront the potential disorder or make full use of the typical interaction pattern to design specific instructional tasks. For example, teachers may appoint moderators who are responsible for managing chats to sort out the viewpoints that have been discussed and present a summary at the end of the discussion, and students can take turns to perform this function across a school term. The intertwined pattern is more suitable for arranging free Q and A sessions with students posting questions and teachers or other students answering questions. The advantage of this arrangement lies in that all the questions, answers and opinions and progress of the discussion are open and transparent to all group members, while students in the offline group discussion are not able to access other groups’ discussion progress. This enables teachers and students to avoid asking or answering similar questions, respectively, and thus improves educational efficiency and equality of information access.

Mobile chat applications like WeChat are widely used for group discussion of a predefined topic or text, which may commonly lead to the appearance of the trunk-branch pattern, referring to multiple responses to a single post. Teachers could also take advantage of the features of this turn-taking pattern for organising a debate or a lead-in of a topic. Both debating and topic lead-in share a similar interaction pattern with one post leading to multiple responses. Again, the summary of the discussion can be arranged for pedagogic needs if the “messiness” of chat text results in confusion of questions and misaligned turns. 

Teachers can also take the sequential movement (i.e., follow a single linear pattern) in their online pedagogical design. They may arrange a storytelling activity by requiring each student to take turns to post one conversational turn only. By the sequential movement one by one, equal opportunity for participation will be guaranteed in this activity. This shows the affordance of mobile text chat as a collaborative tool to fulfil the pedagogic needs of various activities in online contexts. 

Much more research is needed on how online learning tools impact the experience and outcomes of teaching and learning in school contexts. This project has sought to contribute to our understanding of these technologies’ possibilities for learning. Our data shows that users developed genre-specific interaction modes that may be at odds with interaction conventions in formal education environments. The designs for using these tools proposed here may form a starting point for exploring in more detail the possibilities of applying them in online learning contexts. We have deliberately not sought to address the ethical dilemmas surrounding the use of mobile technology, or to comment on issues of access or accessibility. These, too, are areas that should most certainly form a strong line of enquiry in future investigations. Moreover, appropriate visualization will greatly add value to understanding of features of interactional patterns (e.g., the improvement of Figure 3 compared with Figure 2). Visualization of more complex and multimodal online conversations requires further attention from researchers as well. 

## Data Availability

Not applicable.
